# Supplementary material for: Comparative genomics provides new insights into the diversity, physiology, and sexuality of the only industrially exploited tremellomycete: Phaffia rhodozyma
Source: BMC Genomics. 2016 Nov 9;17:901. doi: 10.1186/s12864-016-3244-7 (PMC5103461; doi:10.1186/s12864-016-3244-7)
Supplement: Additional file 6: — List of orphan genes with links to PFAM (related to Additional file 1: Table S1). (ZIP 1428 kb) [file 12864_2016_3244_MOESM6_ESM.zip › PFAM_UNIQUE/G03273_P.html]

#                                                                            --- full sequence --- -------------- this domain -------------   hmm coord   ali coord   env coord
  
# target name        accession   tlen query name           accession   qlen   E-value  score  bias   #  of  c-Evalue  i-Evalue  score  bias  from    to  from    to  from    to  acc description of target
  
#------------------- ---------- ----- -------------------- ---------- ----- --------- ------ ----- --- --- --------- --------- ------ ----- ----- ----- ----- ----- ----- ----- ---- ---------------------
  
G03273\_P             -            893 Activator\_LAG-3      PF11498.3    468    0.0044   14.5  12.0   1   1   1.8e-05    0.0082   13.6  12.0   383   434   472   521   432   545 0.62 - Transcriptional activator LAG-3  
G03273\_P             -            893 DDHD                 PF02862.12   227        10    4.5  12.5   1   2       1.3   5.1e+02   -1.1   0.2    79   124    27    69     7   139 0.55 - DDHD domain  
G03273\_P             -            893 DDHD                 PF02862.12   227        10    4.5  12.5   2   2     0.005         2    6.8   7.7    79   168   439   527   383   542 0.66 - DDHD domain  
G03273\_P             -            893 DUF2722              PF10846.3    417      0.12   10.1   4.7   1   1   0.00066      0.26    8.9   4.7     5    36   487   524   463   583 0.63 - Protein of unknown function (DUF2722)  
G03273\_P             -            893 DUF3451              PF11933.3    225       4.6    5.6  17.1   1   3   0.00037      0.73    8.2   0.1   145   209    12    81     1    94 0.64 - Domain of unknown function (DUF3451)  
G03273\_P             -            893 DUF3451              PF11933.3    225       4.6    5.6  17.1   2   3     0.044        87    1.5   9.2   127   219   430   517   397   523 0.67 - Domain of unknown function (DUF3451)  
G03273\_P             -            893 DUF3451              PF11933.3    225       4.6    5.6  17.1   3   3      0.55   1.1e+03   -2.2   0.1    12    51   790   829   780   838 0.84 - Domain of unknown function (DUF3451)  
G03273\_P             -            893 DUF4482              PF14818.1    141    0.0019   17.6   0.0   1   4         5     6e+03   -3.7   0.3    65    89    53    76    41    97 0.48 - Domain of unknown function (DUF4482)  
G03273\_P             -            893 DUF4482              PF14818.1    141    0.0019   17.6   0.0   2   4       3.4   4.1e+03   -3.0   0.2    89   114   419   444   390   460 0.48 - Domain of unknown function (DUF4482)  
G03273\_P             -            893 DUF4482              PF14818.1    141    0.0019   17.6   0.0   3   4      0.12   1.4e+02    1.8   4.3    73   111   484   522   470   546 0.51 - Domain of unknown function (DUF4482)  
G03273\_P             -            893 DUF4482              PF14818.1    141    0.0019   17.6   0.0   4   4   1.6e-06    0.0019   17.6   0.0    23    72   794   839   787   864 0.83 - Domain of unknown function (DUF4482)  
G03273\_P             -            893 DUF4629              PF15442.1    151       2.2    7.1   6.2   1   1    0.0075       7.4    5.4   6.2    49    95   479   525   469   547 0.49 - Domain of unknown function (DUF4629)  
G03273\_P             -            893 DUF737               PF05300.6    187      0.67    8.6   4.3   1   1    0.0038       1.9    7.1   4.3    43   100   473   519   393   540 0.55 - Protein of unknown function (DUF737)  
G03273\_P             -            893 eIF\_4EBP             PF05456.6    116       2.8    6.4   5.5   1   1    0.0024        14    4.1   5.5    67   110   467   511   460   518 0.58 - Eukaryotic translation initiation factor 4E binding protein (EIF4EBP)  
G03273\_P             -            893 FAM222A              PF15258.1    506       1.6    6.5   5.9   1   1    0.0043       3.7    5.3   5.9   116   336   473   522   391   568 0.60 - Protein family of FAM222A  
G03273\_P             -            893 FCP1\_C               PF09309.5    263      0.47    8.4   5.1   1   1    0.0012       1.5    6.7   5.1   172   247   486   563   474   570 0.57 - FCP1, C-terminal  
G03273\_P             -            893 Fib\_alpha            PF08702.5    146       6.8    5.5   9.9   1   2       2.7   9.4e+02   -1.5   6.8    46    76   487   517   471   540 0.42 - Fibrinogen alpha/beta chain family  
G03273\_P             -            893 Fib\_alpha            PF08702.5    146       6.8    5.5   9.9   2   2    0.0043       1.5    7.6   0.0    45   115   751   823   726   836 0.79 - Fibrinogen alpha/beta chain family  
G03273\_P             -            893 Glutenin\_hmw         PF03157.8    772       3.9    4.3  14.9   1   1    0.0031       6.2    3.6  14.9   159   561   486   517   465   570 0.55 - High molecular weight glutenin subunit  
G03273\_P             -            893 GTSE1\_N              PF15259.1    141     0.085   11.5   3.5   1   1   4.3e-05      0.26    9.9   3.5    63   115   465   517   443   537 0.72 - G-2 and S-phase expressed 1  
G03273\_P             -            893 Hid1                 PF12722.2    895       1.7    4.9   6.7   1   1     0.007       2.8    4.2   6.7   596   701   474   583   432   609 0.49 - High-temperature-induced dauer-formation protein  
G03273\_P             -            893 HlyD\_2               PF12700.2    328      0.24    9.2   2.6   1   1   0.00056      0.48    8.2   2.6    81   141   450   542   381   589 0.65 - HlyD family secretion protein  
G03273\_P             -            893 IncA                 PF04156.9    191      0.13   10.5   8.3   1   2    0.0034      0.33    9.2   4.9    67   127   484   537   430   541 0.69 - IncA protein  
G03273\_P             -            893 IncA                 PF04156.9    191      0.13   10.5   8.3   2   2      0.53        52    2.0   0.1    71   109   800   833   769   862 0.71 - IncA protein  
G03273\_P             -            893 Mnd1                 PF03962.10   188       9.1    4.6   7.6   1   2     0.014       4.2    5.7   1.8    68   106   483   521   446   540 0.70 - Mnd1 family  
G03273\_P             -            893 Mnd1                 PF03962.10   188       9.1    4.6   7.6   2   2      0.19        56    2.0   0.4    79   141   759   818   749   837 0.64 - Mnd1 family  
G03273\_P             -            893 MRP-S25              PF13741.1    231      0.28    9.3   3.4   1   1   0.00027      0.54    8.3   3.4    26    78   467   521   445   536 0.56 - Mitochondrial ribosomal protein S25  
G03273\_P             -            893 NMDAR2\_C             PF10565.4    681      0.69    7.2  11.7   1   2      0.02        29    1.8   6.7   444   483   490   529   474   576 0.74 - N-methyl D-aspartate receptor 2B3 C-terminus  
G03273\_P             -            893 NMDAR2\_C             PF10565.4    681      0.69    7.2  11.7   2   2   0.00038      0.58    7.4   0.2   333   411   762   841   749   847 0.87 - N-methyl D-aspartate receptor 2B3 C-terminus  
G03273\_P             -            893 POX                  PF07526.6    140      0.62    9.0   8.1   1   1   0.00062       1.9    7.4   8.1    35    90   484   539   471   541 0.58 - Associated with HOX  
G03273\_P             -            893 RFX1\_trans\_act       PF04589.8    154       5.1    5.5  11.9   1   1    0.0075        15    4.0  11.9    33    75   473   513   447   529 0.81 - RFX1 transcription activation region  
G03273\_P             -            893 SprA-related         PF12118.3    295         3    5.8  16.9   1   2         8   1.1e+03   -2.7   0.2    28    58    53    69    31   108 0.44 - SprA-related family  
G03273\_P             -            893 SprA-related         PF12118.3    295         3    5.8  16.9   2   2    0.0016      0.23    9.4  11.3    71   114   474   517   374   555 0.41 - SprA-related family  
G03273\_P             -            893 Taxilin              PF09728.4    309       2.5    5.8   9.0   1   2    0.0013       0.8    7.4   4.1    11    74   471   526   466   539 0.62 - Myosin-like coiled-coil protein  
G03273\_P             -            893 Taxilin              PF09728.4    309       2.5    5.8   9.0   2   2      0.21   1.2e+02    0.2   0.2    97   124   795   822   756   833 0.50 - Myosin-like coiled-coil protein  
G03273\_P             -            893 TraG-D\_C             PF12696.2    128       6.5    5.3   7.0   1   2     0.036   1.1e+02    1.4   0.0    36   100     7    74     1    91 0.71 - TraM recognition site of TraD and TraG  
G03273\_P             -            893 TraG-D\_C             PF12696.2    128       6.5    5.3   7.0   2   2     0.019        56    2.3   5.6    70   112   472   514   466   521 0.59 - TraM recognition site of TraD and TraG  
G03273\_P             -            893 YqfQ                 PF14181.1    161       2.7    6.7   5.8   1   2      0.83   1.2e+03   -2.0   0.1   105   130    59    72    29   111 0.47 - YqfQ-like protein  
G03273\_P             -            893 YqfQ                 PF14181.1    161       2.7    6.7   5.8   2   2   0.00067         1    8.1   1.7   100   141   482   518   463   561 0.52 - YqfQ-like protein
